# Impact of social exclusion on salivary progesterone and estradiol in women with borderline personality disorder

**DOI:** 10.1186/s40479-025-00307-1

**Published:** 2025-08-25

**Authors:** Marie Barthauer, Livia Graumann, An Bin Cho, Eugenia Kulakova, Christian Eric Deuter, Oliver T. Wolf, Julian Hellmann-Regen, Stefan Roepke, Christian Otte, Katja Wingenfeld

**Affiliations:** 1 Department of Psychiatry and Psychotherapy, Charité - Universitaetsmedizin Berlin, Corporate Member of Freie Universitaet Berlin, Humboldt, Universitaet zu Berlin and Berlin Institute of Health, Campus Benjamin Franklin, Hindenburgdamm 30, 12203 Berlin, Germany; 2 Deutsches Zentrum für Psychische Gesundheit (DZPG), Berlin, Germany; 3 Institut für Kognitive Neurowissenschaft, Ruhr Universität Bochum, Bochum, Germany

**Keywords:** Borderline personality disorder, Social exclusion, Progesterone, Estradiol

## Abstract

**Background:**

Borderline personality disorder (BPD) is characterized by instability in interpersonal relationships and fear of abandonment, which intensify during stress. Social stressors seem to activate the hypothalamic–pituitary–gonadal (HPG) axis in healthy controls (HC), but this has not been investigated in patients with BPD. This study aimed to investigate the effects of social stress, i.e., social exclusion on changes of progesterone and estradiol levels in women with BPD compared to HC.

**Methods:**

82 women with BPD and 82 HC were randomly assigned to either an exclusion or overinclusion condition of the Cyberball paradigm. Saliva samples were collected at baseline (T1), immediately after Cyberball (T2) and 15 min post-Cyberball (T3). Two 3 × 2 × 2 repeated-measures ANOVAs were conducted with time (T1, T2, T3) as the within-subject factor, and group (BPD vs. HC) and condition (exclusion vs. overinclusion) as between-subject factors.

**Results:**

On progesterone change, the analysis revealed no significant main effects of group or condition, but a significant group × condition interaction. Post-hoc tests showed that within the BPD group, change of progesterone levels at T3 i.e., after Cyberball, were higher after exclusion than overinclusion. For changes of estradiol levels, no significant main effects for group, condition, or their interaction were found.

**Discussion:**

This study provides initial evidence that women with BPD exhibit distinct hormonal dynamics in progesterone after social exclusion versus overinclusion. Further research is needed to better understand this hormonal pattern and its implications for social functioning in BPD.

**Supplementary Information:**

The online version contains supplementary material available at 10.1186/s40479-025-00307-1.

## Introduction

The core symptoms of borderline personality disorder (BPD) involve instability in interpersonal relationships and fear of abandonment [1]. These symptoms often intensify during psychosocial stress, particularly involving social exclusion or (perceived) rejection [2]. Individuals with BPD are more sensitive to experiences of social exclusion and rejection than healthy controls (HC) and tend to exhibit stronger negative emotional reactions in these contexts [3–5]. To experimentally induce feelings of ostracism, several studies used the Cyberball paradigm [6], a virtual ball-tossing game that simulates social inclusion and exclusion by varying the number of ball tosses participants receive from co-players. Patients with BPD consistently perceive exclusion in Cyberball as a strong threat to their social needs and report feeling more excluded, even when being included, compared to HC [5, 7–9].

In response to perceived exclusion, patients with BPD show distinct emotional and physiological reactions, that differ from HC. They tend to show lower emotional empathy for positive emotions, increased other-focused negative emotions, and more hostile-intent attributions compared to HC in response to Cyberball [7, 8, 10–12]. These reactions are often reflected in aggressive but also defensive behaviors including social withdrawal, which contrast with the more adaptive, calm responses seen in HC. This is also reflected in their physiological responses, where patients with BPD have shown lower parasympathetic activity [13–15].

In healthy individuals, Cyberball appears to activate the hypothalamic-pituitary-gonadal (HPG) axis, as suggested by increased progesterone secretion in response to social exclusion in healthy women [16]. Progesterone has been linked to prosocial behavior, enhancing social bonding and interpersonal connections, particularly in stress contexts that include elements of social exclusion [17–19]. Thus, it may play an adaptive role in response to social exclusion. Within the central nervous system (CNS), progesterone is associated with anxiolytic effects by enhancing GABAergic inhibition, promoting calmness, and reducing anxiety [20]. However, not all studies align with this finding, with some reporting no significant effects of Cyberball on progesterone levels in healthy controls [21, 22]. These differences in results compared to the study by Seidel and colleagues (2013) may be due to smaller samples of female participants and methodological differences, such as the timing of hormonal measurements, task design variations, and the methods used to analyze progesterone changes.

In addition to progesterone, few studies have also explored the effects of social exclusion on estradiol in healthy participants, though findings remain inconclusive. Estradiol is discussed as having a positive, potentially protective role in stress regulation through its effects on the hippocampus and prefrontal cortex (for an overview see Albert et al. [23]). However, Weik et al. [24] reported a non-significant drop in plasma estradiol after a public speaking task, possibly due to oral contraceptive use, which can mask natural hormonal responses. Similarly, another study found no estradiol changes in response to the Trier Social Stress Test (TSST; [25]) in a mixed-sex sample (*n* = 39 men, *n* = 44 women) [26]. While the TSST induces performance-based stress and a cortisol spike, Cyberball may uniquely engage pathways related to social pain and exclusion.

Although research directly linking progesterone and estradiol to social exclusion in BPD is lacking, some studies have examined hormonal dynamics of progesterone and estradiol in other contexts. Eisenlohr-Moul et al. [27] investigated naturally occurring hormonal fluctuations in women with BPD and found that symptoms related to interpersonal reactivity, such as irritability, anger, and rejection sensitivity, were more pronounced during the luteal phase, which is characterized by higher progesterone and lower estradiol levels. The same pattern has been shown for increased reactive aggression in BPD [28]. DeSoto et al. [29], however, found that higher estradiol variability, rather than basal levels, was associated with greater symptom severity in BPD. These findings suggest that progesterone and estradiol secretion may contribute to emotional and interpersonal difficulties in BPD. Additionally, a study by Maner et al. [30] showed that individuals with high baseline social anxiety, a feature often observed in BPD, display a decrease in progesterone in response to social exclusion.

The aim of the present analysis, which was part of a larger study [11], was to investigate the effects of social exclusion on hormone secretion of progesterone and estradiol in patients with BPD and healthy controls. We hypothesized that in response to social exclusion progesterone would increase in healthy controls [16] and decrease in women with BPD [30]. We expected no significant changes of estradiol levels in healthy controls following exclusion [24, 26]. In the overinclusion condition, we anticipated no differences in progesterone or estradiol changes between HC and BPD patients. While the impact of estradiol on BPD is inconsistent and data on its role in social exclusion is lacking, we explored the possibility of a decrease in estradiol levels following social exclusion in individuals with BPD. This expectation is based on the known sensitivity of individuals with BPD to social rejection and stress, which often leads to negative mood states. Additionally, lower estradiol levels are linked to increased vulnerability to stress and negative mood states [31, 32].

## Methods

### Participants

Participants were recruited through internet postings, leaflets in hospitals, and the Department for Psychiatry and Psychotherapy at Charité Universitaetsmedizin Berlin. Every participant was provided with both verbal and written details of the procedure and was required to give informed consent before engaging in the study. Participants were compensated 60 Euros, with an additional 30 Euros that could be earned in a computer game [11]. The study was approved by the Ethics Committee at Charité.

The original sample consisted of 98 women with BPD and 98 healthy controls. We excluded all women using hormonal contraception, resulting in 82 naturally cycling women with BPD and 82 healthy controls. Participants were matched for age, education, and menstrual cycle phase. Healthy participants had no lifetime psychiatric diagnoses or treatments. The age range was 18 to 50 years with a BMI between 17.5 and 30. Exclusion criteria included glucocorticoid intake, pregnancy, neurodegenerative, autoimmune, metabolic, endocrine, central nervous system diseases, and other severe somatic diseases. BPD exclusions included acute suicidal behavior, lifetime psychotic symptoms, substance addiction, and an acute major depressive episode. Additionally, individuals taking more than three different psychotropic substances daily or using benzodiazepines were excluded. Dosage stability for at least one week prior to testing was confirmed.

### Procedure

The study comprised two testing sessions. Trained clinicians conducted the Structural Clinical Interview for DSM-5 Disorders (SCID) (German versions of SCID-5-CV, SCID-5-PD; [33]). Participants completed various psychopathology questionnaires on a computer or tablet in a laboratory setting. Detailed information on the specific questionnaires can be found in Graumann et al. [11]. During the second session, mood, wakefulness, and nervousness were assessed using the Multidimensional Mood State Questionnaire (MDMQ; [34]). Subsequently, participants played Cyberball, followed by filling out the MDMQ to measure changes in the variables mentioned above. Participants completed the Need Threat Questionnaire (NTQ; [35]) to assess need threat related to Cyberball. Additionally, ostracism intensity was assessed with two items *(“I felt ignored”* and *“I felt rejected”)* and a sum score was calculated(see detailed description below). Additionally, participants were asked to estimate the percentage of ball tosses they received during the Cyberball game. We also assessed the extent to which participants believed they were playing the game with real players. Following the Cyberball task, participants completed computer-based tests, which have been reported elsewhere [11, 36].

### Cyberball: manipulation check and need threat

Participants were asked to estimate the percentage of ball tosses they received during the Cyberball game: “If the game is fair, meaning the ball is thrown equally often to each player, then (with three players in total) each player receives 33% of the throws. How many percent of the throws did you receive?”. Participants rated their perceived ostracism intensity using two items (“I felt ignored” and “I felt rejected”) on a 5-point Likert scale. The total ostracism score was calculated by summing these two ratings (range: 2–10). Their belief in the cover story was also assessed using a Likert scale (1 = “Not at all,” 2 = “A little,” 3 = “Moderately,” 4 = “Completely”). This was done analogously to the study by Weinbrecht et al. [8].

Need threat in response to Cyberball was assessed using the German version of the Need Threat Questionnaire (NTQ [35]), which was originally developed by Williams et al. [6]. Participants rated their agreement with 14 statements on a 5-point Likert scale (1 = not at all, 5 = completely), covering four subscales: belonging (e.g., *“I felt rejected”*), control (e.g., *“I felt powerful”*), self-esteem (e.g., *“I felt popular”*), and meaningful existence (e.g., *“I felt non-existent”*). In line with Gutz et al. [7], a total need threat score was calculated by summing all four subscales (range: 4–20), with higher scores reflecting greater perceived threat to social, cognitive, and emotional needs.

### Multidimensional mood state questionnaire (MDMQ)

Participants rated their mood, nervousness, and wakefulness on a 5-point Likert scale (1 = not at all, 5 = completely). Subscale scores were calculated by summing the respective items, resulting in a total score ranging from 4 to 20, with lower scores indicating negative mood, greater tiredness, and more nervousness.

### Social exclusion

Participants were randomized into either an exclusion or an overinclusion condition of the Cyberball paradigm [37]. The overinclusion condition was chosen as the control condition due to biased perception of inclusion in patients with BPD [8, 38]. Each condition lasted two to three minutes with 30 ball tosses. In the exclusion condition, participants received the ball twice within the first six throws, then not again. In the overinclusion condition, participants received 45% of all throws. Participants were instructed to visualize the experience as an authentic interaction with real players. The instructions explicitly conveyed that they would be participating in a virtual ball-tossing activity with two co-players through an internet connection, notwithstanding the fact that these co-players were computer-generated. All participants were debriefed after the experiment.

### Physiological outcome variables

For analyses of progesterone and estradiol, saliva samples were collected using SaliCap devices (IBL, Hamburg, Germany) at the following time points: baseline (T1 = 0 min), immediately after Cyberball (T2 = 20 min after baseline), and 15 min after Cyberball (T3 = 35 min after baseline). The timing between T1 and T2 reflects the duration of post-baseline activities, including administration of the MDMQ, transition to the computer station, instructions, and the Cyberball task (~ 3–4 min), after which the second saliva sample (T2) was taken. Samples were stored at − 80 °C until biochemical analyses, performed at the Neurobiology Laboratory of the Department of Psychiatry and Psychotherapy, Charité, Universitaetsmedizin Berlin. Progesterone and estradiol levels were determined using a competitive immunoassay (IBL/TECAN, Hamburg, Germany). The limit of detection was 3.31 pg/ml for progesterone and 2.1 pg/ml for estradiol. Prediction parameters (coefficient of variation; CV) for medium concentrations at 50 pg/ml averaged below 5% for intra-assay and below 10% for inter-assay variance.

### Statistics

Statistical analyses were conducted using SPSS version 29. ANOVAs were used to compare the study groups (BPD-Overinclusion, BPD-Exclusion, HC-Overinclusion, HC-Exclusion) with respect to demographic variables (age, body mass index, years of school education), baseline progesterone and estradiol levels, psychopathology measures (BDI-II, BSL-23, ITQ, RSQ), and baseline mood states (good/bad, awake/tired, calm/nervous). Categorical variables (smoking status, menstrual cycle phase) were analyzed by chi-square (χ²) tests.

We conducted a series of 2 × 2 ANOVAs with group (BPD vs. HC) and condition (exclusion vs. overinclusion) as between-subject factors. These analyses were applied to the estimated percentage of ball tosses received, ostracism intensity and scores on the Need Threat Questionnaire (NTQ). NTQ analyses were applied separately, for the total need threat score and the four subscales: belonging, control, self-esteem, and meaningful existence. Mood ratings were analyzed using a 2 × 2 × 2 ANOVA with time (T1, T2) as a within-subject factor, and group (BPD vs. HC) and condition (overinclusion vs. exclusion) as between-subject factors. The three subscales of the Multidimensional Mood State Questionnaire (MDMQ), good–bad, awake–tired, and calm–nervous, were analyzed separately.

To analyze the effects of social exclusion vs. overinclusion on hormonal changes, we conducted a 3 × 2 × 2 rmANOVA with time (T1, T2, T3) as the within-subjects factor, and group (BPD vs. HC) and condition (exclusion vs. overinclusion) as between-subjects factors. Given the high interindividual variability in progesterone and estradiol levels, we baseline-corrected all values by setting T1 to 0 and subtracting T1 from T2 and T3 for each participant. This approach allowed us to focus on the relative hormonal change rather than absolute hormone levels, improving comparability across participants. Post-hoc tests were conducted when interaction effects were significant. For progesterone, we additionally, we conducted a 2 × 2 ANOVA using change score (T3–T1) as the dependent variable. We also ran an ANCOVA with T1 as covariate to account for baseline hormone levels when analyzing this change score. Correlations were conducted to examine associations between baseline-corrected hormone levels (progesterone, estradiol) at T3 and self-reported mood and need threat (MDMQ, NTQ) assessed after the Cyberball task.

## Results

### Demographic and clinical data

In the exclusion condition, there were 44 women with BPD and 45 HC. In the overinclusion condition, there were 38 women with BPD and 37 HC. There were no significant group differences in terms of age, years of school, body mass index, phase of the menstrual cycle, and baseline estradiol values. There were more smokers across both conditions in the BPD group than in the HC groups (all *ps* < 0.001) and progesterone values differed significantly between the groups, with the HC group in the overinclusion condition having significantly lower values than the other groups. For results, see Table 1.

**Table 1:** Sample characteristics, psychopathology questionnaire data, mood and baseline hormone levels

Variable	BPD-Overinclusion *n* = 38	BPD-Exclusion *n* = 44	HC-Overinclusion *n* = 37	HC-Exclusion *n* = 45	Statistics
Age (M/SD)	27.97 (6.35)	29.00 (8.43)	27.76 (6.69)	27.69 (6.12)	*F*(3, 160) = 0.32, *p* =.81, *η*^2^ = 0.01
Years of school education (M/SD)	11.68 (1.09)	11.75 (1.33)	11.89 (1.05)	12.13 (0.97)	*F*(3, 160) = 0.32, *p* =.81, *η*^2^ = 0.03
Smoker (y/n)	(20/18)	(17/27)	(7/30)	(4/41)	χ^*2*^(3) = 22.83, *p* <.001*
Body mass index (M/SD)	23.14 (3.53)	22.65 (3.00)	22.09 (2.39)	21.65 (2.61)	*F*(3, 160) = 2.06, *p* =.11, *η*^2^ = 0.04
Cycle phase (follicular/luteal/no cycle)	(13/24/0)	(13/26/4)	(14/23/0)	(16/27/2)	χ^2^(6) = 6.90, *p* =.33
BDI-II (M/SD)	26.95 (11.56)	27.51 (12.74)	1.38 (1.69)	1.71 (2.47)	*F*(3, 159) = 117.48, *p* <.001**, *η*^2^ = 0.69
BSL-23 (M/SD)	1.89 (0.95)	1.92 (0.84)	0.06 (0.07)	0.11 (0.15)	*F*(3, 160) = 112.73, *p* <.001**, *η*^2^ = 0.68
ITQ (M/SD)	11.16 (5.73)	9.80 (6.57)	0.89 (1.76)	0.82 (1.68)	*F*(1, 160) = 182.23, *p* <.001**, *η*^2^ = 0.53
RSQ (M/SD)	18.28 (7.25)	19.08 (6.12)	5.66 (2.40)	6.39 (2.94)	*F*(3, 160) = 61.22, *p* <.001**, *η*^2^ = 0.53
Baseline progesterone (T1) (M/SD)	98.04 (84.97)	100.72 (117.50)	63.15 (59.78)	116.13 (132.33)	*F*(3, 151) = 3.95, *p* =.05***, *η*^2^ = 0.06
Baseline estradiol (T1) (M/SD)	4.46 (2.16)	4.14 (1.69)	4.28 (1.91)	3.86 (1.44)	*F*(3, 156) = 0.81, *p* =.49, *η*^2^ = 0.02

*BPD* borderline personality disorder, *HC* healthy controls, *n* sample size, *M* mean, *SD* standard deviation, *y* yes, *n* no, *BDI-II* Beck Depression Inventory, *BSL-23* Borderline Symptom List - short version, *ITQ* International Trauma Questionnaire, *RSQ* Rejection Sensitivity Questionnaire

*Post-hoc tests revealed significant differences between BPD and HC

**BPD-Overinclusion = BPD-Exclusion > HC-Overinclusion = HC-Exclusion

***The significant ANOVA is driven by lower progesterone values in HC-Overinclusion than BPD-Overinclusion, BPD-Exclusion and HC-Exclusion

Of the BPD group, 37 women were inpatients and 51 were outpatients. Forty-two women with BPD were free of psychotropic medication, and 40 reported medication use. The most frequent comorbid diagnosis in BPD was PTSD *n* = 20. For further details on medication and comorbid diagnoses, see supplementary material. Women with BPD had significantly higher scores on all self-report questionnaires for clinical symptoms, rejection sensitivity, as well as lower mood, greater tiredness, and greater nervousness, all *p*s < 0.001. In addition, the BPD-Overinclusion and BPD-Exclusion groups did not differ in baseline levels of mood, tiredness, and nervousness (all *p*s > 0.05). For results, see Table 1.

### Cyberball: manipulation check, need threat and mood

As a manipulation check, we analyzed estimated percentages of received ball tosses. The analysis showed no effect of group (*F*(1, 158) = 1.35, *p* =.25, *η*^2^ = 0.01). However, there was a significant effect of Cyberball condition with a large effect size (*F*(1, 158) = 421.28, *p* <.001, *η*^2^ = 0.73), with higher change in values for overinclusion vs. exclusion. There was no significant interaction effect. Both groups accurately estimated the ball possession during the Cyberball conditions. Women with BPD believed the cover story more than HC did. For ostracism intensity, we found significant main effects of group (*F*(1, 160) = 18.48, *p* <.001, *η*² = 0.10) and condition (*F*(1, 160) = 369.96, *p* <.001, *η*² = 0.70), indicating that participants with BPD and those in the exclusion condition felt more ostracized.

Analysis of total need threat values showed a main effect of group (*F*(1, 160) = 73.9, *p* <.001, *η*^*2*^ = 0.32) and condition *(F*(1, 160) = 114.19, *p* <.001, *η*^*2*^ = 0.42). Total need threat was higher in women with BPD vs. HC, and higher after exclusion vs. overinclusion. We also found a significant interaction effect (*F*(1, 160) = 3.77, *p* =.05, *η*^*2*^ = 0.02). This indicates that participants with BPD reported stronger threat to fundamental needs compared to HC following both Cyberball conditions, with more pronounced threat following exclusion (*t*(87) = 7.29, *p* <.001) than after overinclusion (*t*(73) = 4.96, *p* <.001). Analysis of the NTQ subscales revealed significant group × condition interactions for belonging (*F*(1,160) = 4.93, *p* =.03, *η*² = 0.03) and meaningful existence (*F*(1,160) = 15.12, *p* <.001, η² = 0.09), indicating that the overall interaction in total need threat was primarily driven by stronger threat to these two fundamental needs in the BPD group, particularly after exclusion. Mean values and statistics of NTQ are reported in Table 2. On the Multidimensional Mood State Questionnaire (MDMQ), women with BPD reported worse mood, feeling more tired, and more nervous than HC after both Cyberball conditions, all *p*s < 0.001. There were no significant group × condition interaction effects. Mean values and statistics of the MDMQ are reported in Table 3.

**Table 2:** Cyberball manipulation check: need threat questionnaire results, ostracism intensity, perceived ball tosses and belief cover story

Variable	Group	Condition	M	SD	Statistics
NTQ Overall Need threat	BPD	Overincl.	11.67	3.08	Group: *F*(1,160) = 73.9, *p* <.001, *η*^*2*^ = 0.32
		Exclusion	17.09	2.89	Condition: *F*(1,160) = 114.19, *p* <.001, *η*^*2*^ = 0.42
	HC	Overincl.	8.95	1.56	Group × condition: *F*(1,160) = 3.77, *p* =.05, *η*^*2*^ = 0.02
		Exclusion	12.64	2.87	
NTQ Belonging	BPD	Overincl.	2.30	1.07	Group: *F*(1,160) = 25.18, *p* <.001, *η²* = 0.14
		Exclusion	4.10	0.98	Condition: *F(*1,160) = 92.97, *p* <.001, *η²* = 0.37
	HC	Overincl.	1.87	0.64	Group × condition: *F*(1,160) = 4.93, *p* =.03, *η²* = 0.03
		Exclusion	3.00	1.08	
NTQ Control	BPD	Overincl.	4.27	0.89	Group: *F*(1,160) = 5.29, *p* =.02, *η²* = 0.03
		Exclusion	4.60	0.72	Condition: *F*(1,160) = 14.23, *p* <.001, *η²* = 0.08
	HC	Overincl.	3.86	0.83	Group × condition: *F*(1,160) = 1.21, *p* =.27, *η²* = 0.01
		Exclusion	4.45	0.69	
NTQ Self-esteem	BPD	Overincl.	3.45	1.10	Group: *F*(1,160) = 115.31, *p* <.001, *η²* = 0.42
		Exclusion	4.44	0.74	Condition: *F*(1,160) = 49.63, *p* <.001, *η²* = 0.24
	HC	Overincl.	2.00	0.76	Group × condition: *F*(1,160) = 0.04, *p* =.85, *η²* = 0.00
		Exclusion	2.94	0.86	
NTQ Meaningful existence	BPD	Overincl.	1.75	0.99	Group: *F*(1,160) = 53.46, *p* <.001, *η²* = 0.25
		Exclusion	3.95	1.14	Condition: *F*(1,160) = 113.23, *p* <.001, *η²* = 0.41
	HC	Overincl.	1.23	0.57	Group × condition: *F*(1,160) = 15.12, *p* <.001, *η²* = 0.09
		Exclusion	2.25	1.04	
Ostracism Intensity	BPD	Overincl.	2.97	1.14	Group: *F*(1, 160) = 18.48, *p* <.001, *η*^*2*^ *=.*10
		Exclusion	8.57	1.91	Condition: *F*(1,160) = 369.96, *p* <.001, *η*^*2*^ = 0.70
	HC	Overincl.	2.19	0.74	Group × condition: *F*(1,160) = 1.97, *p* =.16, *η*^*2*^ = 0.01
		Exclusion	7.02	2.01	
Perceived amount of ball tosses (%)	BPD	Overincl.	52.61	21.88	Group: *F*(1, 158) = 1.35, *p* =.25, *η²* = 0.01
		Exclusion	7.05	7.01	Condition: *F*(1, 158) = 421.28, *p* <.001, *η²* = 0.73
	HC	Overincl.	49.14	28.17	Group × condition: *F*(1, 158) = 0.80, *p* =.37, η² = 0.01
		Exclusion	6.47	49.14	
Belief Cover Story	BPD	Overincl.	2.22	1.03	Group: *F*(1,157) = 4.77, *p* =.03, *η*^*2*^ = 0.03
		Exclusion	1.64	0.69	Condition: *F*(1,157) = 13.401, *p* <.001, *η*^*2*^ = 0.08
	HC	Overincl.	1.84	0.87	Group × condition: *F*(1,157) = 0.33, *p* =.57, *η*^*2*^ = 0.00
		Exclusion	1.42	0.58	

*BPD* borderline personality disorder, *HC* healthy controls, *Overincl* Cyberball overinclusion condition, *M* mean, *SD* standard deviation, *NTQ* Need Threat Questionnaire

**Table 3:** Results of the multidimensional mood state questionnaire (MDMQ)

MDMQ Scale	Group	Condition	Time	M	SD	Statistic
Good versus bad	BPD	Overincl.	Before	12.79	3.10	Between-subjects
			After	12.58	3.67	Group: *F*(1,159) = 282.69, *p* <.001***, *η*^*2*^ *=* 0.62
		Excl.	Before	12.53	3.52	Condition: *F*(1,159) = 1.46, *p* =.31, *η*^*2*^ *=* 0.01
			After	11.37	3.49	Group × condition: *F*(1,159) = 0.61, *p* =.44, *η*^*2*^ = 0.01
	HC	Overincl.	Before	18.59	0.90	Within subjects
			After	18.54	1.19	Time: *F*(1,159) = 4.14, *p* =.04*, *η*^*2*^ *=* 0.03
		Excl.	Before	18.47	1.60	Time × group: *F*(1,159) = 2.55, *p* =.11, *η*^*2*^ *=* 0.02
			After	18.36	1.63	Time × condition: *F*(1,159) = 1.78, *p* =.18, *η*^*2*^ *=.*01
						Time × group × condition: *F*(1,159) = 1.40, *p* =.24, *η*^*2*^ *=.*01
Awake versus tired	BPD	Overincl.	Before	11.45	3.76	Between-subjects
			After	11.03	3.33	Group: *F*(1,159) = 133.67, *p* <.001***, *η*^*2*^ = 0.46
		Excl.	Before	10.84	3.35	Condition: *F*(1,159) = 3.53, *p* =.06, *η*^*2*^ = 0.02
			After	9.91	3.07	Group × condition: *F*(1,159) = 0.00, *p* =.98, *η*^*2*^ = 0.00
	HC	Overincl.	Before	16.41	2.51	Within subjects
			After	16.51	2.64	Time: *F*(1,159) = 4.35, *p* =.04*, *η*^*2*^ *=* 0.03
		Excl.	Before	15.76	2.84	Time × group: *F*(1,159) = 2.72, *p* =.10, *η*^*2*^ *=* 0.02
			After	15.49	3.12	Time × condition: *F*(1,159) = 1.49, *p* =.22, *η*^*2*^ *=.*01
						Time × group × condition: *F*(1,159) = 0.04, *p* =.85, *η*^*2*^ *=.*00
Calm versus nervous	BPD	Overincl.	Before	11.61	3.05	Between-subjects
			After	11.82	3.93	Group: *F*(1,159) = 224.84, *p* <.001***, *η*^*2*^ = 0.59
		Excl.	Before	10.02	3.44	Condition: *F*(1,159) = 5.15, *p* =.03*, *η*^*2*^ = 0.03
			After	10.72	3.51	Group x condition: *F*(1,159) = 0.76, *p* =.39, *η*^*2*^ *=* 0.01
	HC	Overincl.	Before	17.86	2.00	Within subjects
			After	17.59	2.34	Time: *F*(1,159) = 1.98, *p* =.16, *η*^*2*^ *=* 0.01
		Excl.	Before	16.91	2.45	Time × group: *F*(1,159) = 0.91, *p* =.34, *η*^*2*^ *=* 0.01
			After	17.36	2.52	Time × condition: *F*(1,159) = 2.44, *p* =.12, *η*^*2*^ *=.*01
						Time × group × condition: *F*(1,159) = 0.09, *p* =.76, *η*^*2*^ *=.*00

*MDMQ* Multidimensional Mood State Questionnaire, *BPD* borderline personality disorder, *HC* healthy controls, *M* mean, *SD* standard deviation

We did not find any significant correlations between the self-reported measures of mood and need threat (MDMQ, NTQ) and change in progesterone or estradiol at T3.

## Main Results

### Progesterone

The 3 × 2 × 2 rmANOVA with time (T1, T2, T3) as a within-subjects factor and group (BPD, HC) and condition (overinclusion, exclusion) as between-subjects factors, did not reveal a significant main effect of time (*F*(2, 149) = 2.52, *p* =.08, *η*^2^ = 0.02). Additionally, neither the time × group interaction (*F*(2, 149) = 0.48, *p* =.62, *η²* = 0.00) nor the time × condition interaction (*F*(2, 149) = 1.22, *p* =.30, *η²* = 0.01) was significant. The time × group × condition interaction was also not significant (*F*(2, 149) = 2.69, *p* =.07, *η²* = 0.02). 

**Fig. 1:**
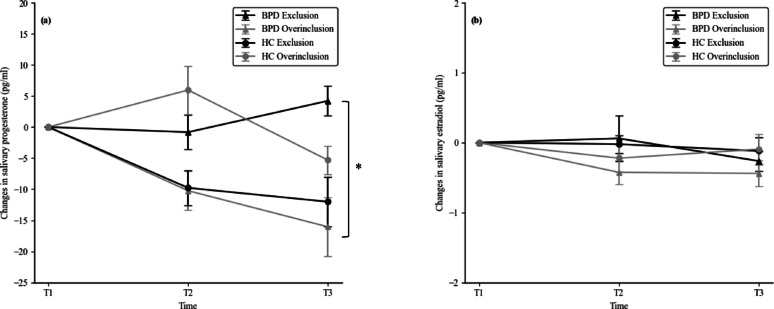
Mean (± SEM) change score of salivary progesterone (**a**) and estradiol (**b**) levels (pg/ml), baseline-corrected, at three time points: T1 (baseline), T2 (immediately after Cyberball), and T3 (15 min post-Cyberball), shown separately by group (BPD vs. HC) and condition (exclusion vs. overinclusion). A significant group × condition interaction was found for progesterone (*p* =.03). Post-hoc comparison indicated that within the BPD group, change of progesterone values at T3 were significantly higher following exclusion than overinclusion (*p* =.03).

Regarding between-subject effects, there was no significant effect of group (*F*(1, 150) = 0.09, *p* =.76, *η*^2^ = 0.00) or condition (*F*(1, 150) = 0.01, *p* =.94, *η*^2^ = 0.00). However, a significant group × condition interaction was found (*F*(1, 150) = 4.85, *p* =.03, *η*^2^ = 0.03). Post-hoc analysis suggested that within the BPD group, progesterone changes at T3, i.e., after Cyberball, were higher in the exclusion condition (*M* = 4.22, *SD* = 30.56), compared to the overinclusion condition (*M* = − 17.83, *SD* = 55.60) (*t*(75) = − 2.20, *p* =.03). Results are presented in Fig. 1. 

**Fig. 2:**
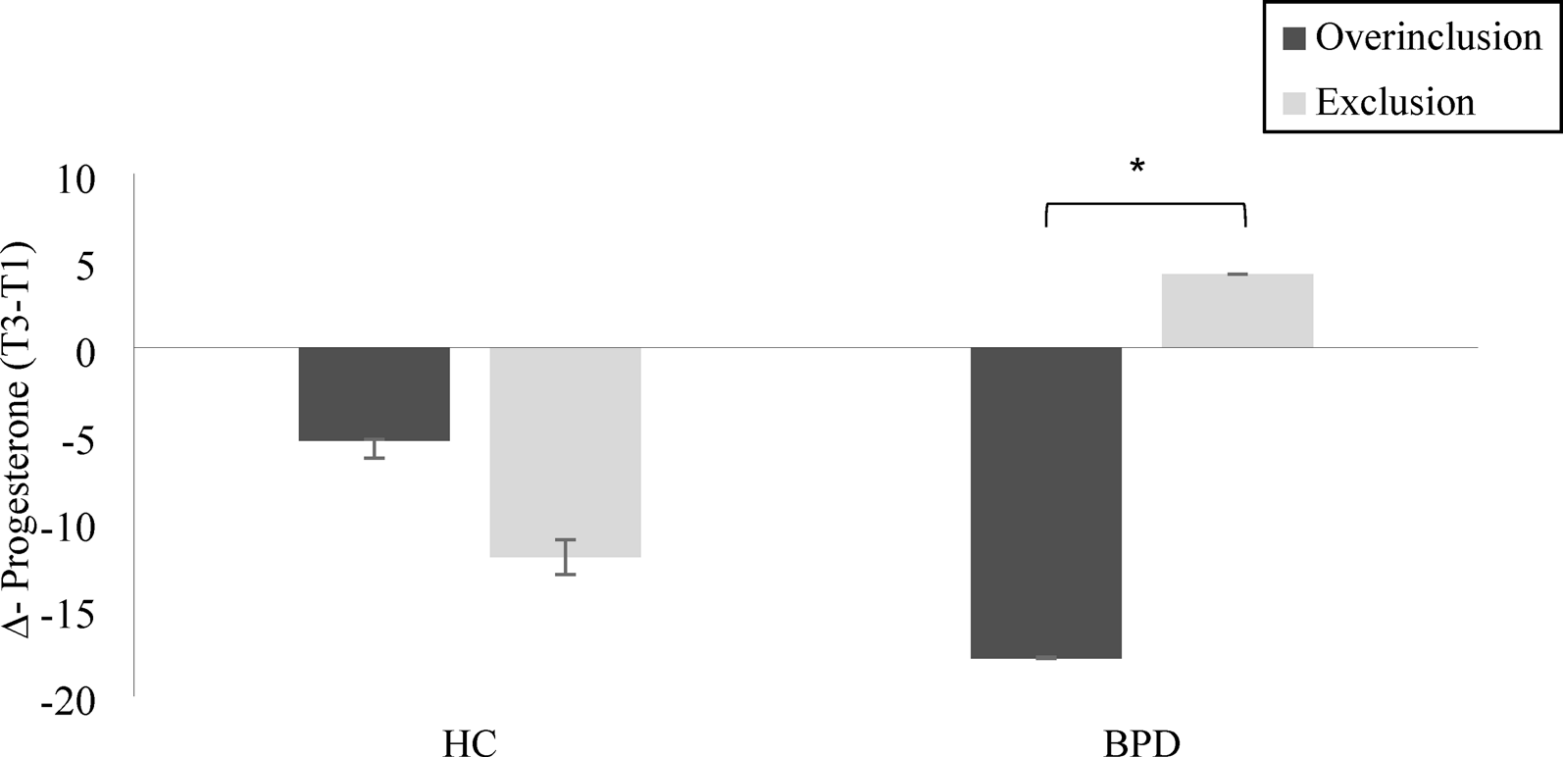
Mean (± SEM) change score (Δ) of salivary progesterone (pg/ml) from T1 (before Cyberball overinclusion or exclusion) to T3 (15 min after Cyberball) in women with borderline personality disorder (BPD) and healthy controls (HC). A significant group × condition interaction was found (*p* =.04). Error bars represent standard error of the mean

We additionally conducted a 2 × 2 ANOVA with change score (T3–T1) as outcome, which confirmed the group × condition interaction (*F*(1,151) = 4.19, *p* =.04, η² = 0.03). There were no main effects of group (F(1, 151) = 0.07, *p* =.79, η² = 0.00), or condition (F(1, 151) = 1.20, *p* =.28, η² = 0.01) (Fig. 2). When including T1 as covariate in an ANCOVA, the interaction was not significant (*p* =.12), but a condition effect emerged (*p* =.05), with higher estimated marginal means for exclusion (*M* = 2.67, *SE* = 5.32) than overinclusion (*M* = − 7.70, *SE* = 5.60).

### Estradiol

The 3 × 2 × 2 rmANOVA with time (T1, T2, T3) as a within-subjects factor, and group (BPD, HC) and condition (overinclusion, exclusion) as between-subjects factors, did not reveal a significant main effect of time (*F*(2, 149) = 2.49, *p* =.08, η² = 0.02). Additionally, neither the time × group interaction (*F*(2, 155) = 0.75, *p* =.47, *η²* = 0.01) nor the time × condition interaction (*F*(2, 155) = 1.49, *p* =.23, *η²* = 0.01) was significant. The time × group × condition interaction was also not significant (*F*(2, 155) = 0.25, *p* =.78, *η²* = 0.00).

Regarding between-subjects effects, there were no significant main effects observed for group (*F*(1, 150) = 0.75, *p* =.39, η² = 0.01) or condition (*F*(1, 150) = 1.42, *p* =.24, η² = 0.01). Additionally, the group × condition interaction was not significant (*F*(1, 150) = 0.48, *p* =.49, η² = 0.00). Results are presented in Fig. 1.

The inclusion of BMI and menstrual cycle phase as covariates in the analysis of both progesterone and estradiol did not change the significance of the findings.

## Discussion

This study examined the effects of social exclusion and overinclusion on changes of progesterone and estradiol levels in women with BPD compared to healthy women. For progesterone, a significant interaction between group and condition was observed, with post-hoc tests suggesting that, within the BPD group, changes of progesterone values differed between the overinclusion and exclusion conditions following the Cyberball task. Healthy controls showed no significant differences in progesterone changes between conditions. For estradiol, no significant main or interaction effects were observed. Below, we discuss these findings, focusing first on progesterone, followed by estradiol.

### Progesterone

We hypothesized that healthy controls would show an increase in progesterone following social exclusion, consistent with a previous study [16]. However, our results did not support this hypothesis. No significant changes in progesterone were observed in neither the exclusion nor the overinclusion condition in HC. In our sample, healthy women did not report strong negative emotional reactions to Cyberball, as they did not feel notably nervous, tired, or bad after either condition. This suggests that the Cyberball paradigm may not have been sufficiently impactful to elicit a measurable response of progesterone. Our results replicate previous findings that show an absence of significant hormonal responses in progesterone and cortisol within the HC group [21, 22]. This absence may be due to the Cyberball paradigm’s lack of face-to-face interaction, which reduces the immediacy of the social threat. Additionally, the Cyberball paradigm does not require active planning, physical or cognitive engagement, which are factors that seem to activate the HPA and HPG Axis [21].

Our findings diverge from results of Seidel et al. [16]. A few methodological differences may explain this discrepancy. First, the authors used a block design with alternating inclusion and exclusion phases in the experimental condition, which may create a more pronounced social contrast and influence progesterone secretion. Second, our hormonal measurements were taken at baseline, immediately after, and 15 min post-task, whereas their sampling occurred approximately 20 min after Cyberball onset, suggesting our timing may have been too early to capture significant changes. The difference in the results may additionally be due to differences in the sample. Third, while the study of Seidel et al. [16] included both men and women, our research involved a larger, exclusively female sample of 82 women with BPD and 82 healthy controls. This allows for a more reliable analysis of hormonal patterns specific to women in our study.

For women with BPD, we hypothesized a decrease in progesterone following social exclusion. This was based on prior research showing a drop in progesterone in individuals with high social anxiety and evidence of withdrawal or socially anxious behavior in BPD patients [30, 39, 40]. While our findings did not support this hypothesis, we observed that progesterone change scores at T3, i.e., after Cyberball, were higher in participants with BPD who were excluded compared to those who were overincluded.

In line with previous studies, our results suggest that women with BPD perceive and process the Cyberball task with a heightened sensitivity to social rejection compared to healthy controls [5, 7–9]. This is reflected in significantly higher reports of need threat in the BPD group, particularly after exclusion, but also elevated compared to HC following overinclusion. In addition, women with BPD reported stronger feelings of ostracism, more negative mood, increased tiredness, and greater nervousness across both conditions. They were also more likely than HC to believe the cover story, suggesting they experienced the Cyberball game as more realistic and personally involving. These patterns are consistent with prior research showing that individuals with BPD often feel excluded even under fair inclusion, and that only overinclusion reduces, but does not eliminate, perceived social threat [8, 38, 41]. However, the observed difference in progesterone change scores at T3 between exclusion and overinclusion could possibly suggest that exclusion may still hold distinct relevance on an endocrinological level, despite similarly elevated subjective reports. We can only speculate that this pattern may reflect that overinclusion is perceived as socially inadequate, while exclusion more strongly engages physiological systems linked to social stress or affiliative motivation—systems that progesterone has previously been linked to [17, 18, 42]. This group-specific difference between conditions was also evident when analyzing change scores in a 2 × 2 ANOVA. When baseline progesterone (T1) was included as a covariate, the interaction attenuated, though a main effect of condition emerged. This analytic pattern underscores how baseline adjustment influences the detection of differences in gonadal hormone changes, while still suggesting that progesterone change scores were higher following social exclusion compared to overinclusion.

### Estradiol

In contrast to progesterone, our analysis of estradiol revealed no significant interaction effects or main effects of group or condition. Both the BPD and HC groups showed no significant changes in estradiol levels from T1 to T3, regardless of whether they experienced social exclusion, or overinclusion. This is consistent with previous studies that reported no significant stress-induced changes in estradiol levels during social stress tasks in healthy individuals [24, 26, 43]. In BPD, this may indicate that estradiol is less responsive to short-term social stressors like Cyberball, particularly in comparison to progesterone. In animal studies, estradiol appears to be more stable and might be more responsive to physical stressors, such as sleep deprivation, footshock, or swim stress, which involve substantial physiological demands [44, 45]. Additionally, estradiol synthesis is more complex than progesterone, involving several enzymatic steps [46], thus the timing of our measurements may not have captured peak changes in estradiol levels, which could have occurred at different intervals post-stressor. Estradiol’s role in social stress responses may also be more complex and influenced by chronic stress, changes in brain structures, and sleep patterns, which were not accounted for in this study [47, 48].

### Strengths and limitations

The study has several strengths and limitations. First, the artificial laboratory setting may not fully capture the complexities of real-world social interactions. Additionally, participants were tested across different menstrual cycle phases, however, including this factor in the analysis did not alter the results. Some BPD patients were taking psychotropic medication or had comorbidities such as PTSD or anxiety disorders, which may have influenced hormonal responses. We used baseline-corrected hormone values (i.e., change scores) to account for baseline differences and intraindividual variability, especially in progesterone. This should be considered when interpreting the findings. Moreover, this study focused on women, as BPD is more prevalent in women, limiting the generalizability of the findings to men. Another important limitation of our study is the absence of a long inclusion phase preceding the exclusion condition, which some studies suggest may impact the effects of exclusion, potentially influencing participants’ responses. In addition, the absence of a fair inclusion condition limits the ability to determine whether the observed hormonal differences are specific to exclusion, as fair inclusion has also been shown to elicit atypical responses in individuals with BPD. One strength of the study was the exclusion of individuals with major depressive episodes, due to variations in the functioning of the hypothalamic-pituitary-adrenal axis between those with and without a depressive episode in patients with BPD [49, 50]. Furthermore, the exclusion of participants using hormonal contraception ensured that natural progesterone and estradiol secretion were measured. We also excluded individuals with a BMI under 17.5 or over 30, as weight can influence the synthesis of these hormones.

### Conclusion and future direction

In conclusion, our findings provide initial evidence that in women with BPD, but not in healthy women, social exclusion may influence progesterone secretion differently than overinclusion. While the underlying mechanisms remain unclear, one possible conceptual explanation, requiring further investigation, is that exclusion may carry distinct biological relevance in BPD. Given progesterone’s previously described role in affiliative motivation and stress regulation, this pattern could reflect differential engagement of systems related to social bonding or affective modulation. Clinically, these results underscore the importance of considering both subjective and endocrinological factors, specifically gonadal hormonal dynamics, when investigating interpersonal sensitivity in BPD. Further research is needed to determine whether such hormonal differences are consistent, meaningful, and relevant to the interpersonal difficulties characteristic of the disorder. Future studies should incorporate an additional fair inclusion condition to capture the full dynamics of social exclusion effects. Furthermore, the impact of progesterone should be investigated further, for example by exploring whether pharmacologically increasing progesterone in BPD could modulate responses to social exclusion and overinclusion. Additionally, research could examine progesterone’s role in emotional regulation using methods that more closely mimic real-life social interactions. Integrating self-reported assessments of social affiliation, with objective hormonal measures and implicit affiliation tasks could clarify the behavioral function of progesterone. Finally, fMRI studies could map how varying progesterone levels affect brain areas involved in social processing, offering deeper insights into how progesterone modulates social cognition and behavior in BPD.

Future research should focus on more realistic social exclusion paradigms and longitudinal designs to track hormonal changes over time in response to repeated social stressors, offering deeper insights into the regulation of hormonal responses in women with BPD.

## Electronic supplementary material

Below is the link to the electronic supplementary material.

Supplementary Material 1

## Data Availability

The authors confirm that the data supporting the findings of this study are available within the article and its supplementary materials. Further data are available from corresponding author (KW) upon reasonable request.
