# Supplementary material for: Impact of social exclusion on salivary progesterone and estradiol in women with borderline personality disorder
Source: Borderline Personal Disord Emot Dysregul. 2025 Aug 25;12:32. doi: 10.1186/s40479-025-00307-1 (PMC12376725; doi:10.1186/s40479-025-00307-1)
Supplement: Supplementary file 1 — Supplementary Material 1 [file 40479_2025_307_MOESM1_ESM.docx]

**Supplementary Material**

**Results**

**Demographics**

Of the BPD group, 22 individuals reported using one substance, 15 using two substances, and three using three different substances. The substances reported are listed in Table 1, while comorbid diagnoses within the BPD group are presented in Table 2.

Table 1 Psychopharmacological use in the BPD group

| Substance | Number of Patients |
| --- | --- |
| Serotonin and noradrenaline reuptake inhibitors (SNRI) | *n* = 14 |
| Selective serotonin reuptake inhibitors (SSRI) | *n* = 15 |
| Anti-psychotics | *n* = 12 |
| Dopamine and noradrenergic reuptake inhibitors (NDRI) | *n* = 3 |
| Tricyclic antidepressants | *n* = 3 |
| Noradrenergic and specific serotonergic antidepressants (NaSSa) | *n* = 2 |
| Anticonvulsants | *n* = 2 |
| Methylphenidate | *n* = 2 |

Table 2 Comorbid diagnoses in the BPD Group

| Diagnosis | Number of Patients |
| --- | --- |
| Eating Disorder | *n* = 18 |
| PTSD | *n* = 20 |
| Alcohol abuse | *n* = 5 |
| Drug abuse | *n* = 5 |
| Agoraphobia with Panic Disorder | *n* = 4 |
| Social Phobia | *n* = 3 |
| ADHD | *n* = 3 |
| Panic Disorder | *n* = 3 |
| Obsessive-Compulsive Disorder | *n* = 3 |
| Agoraphobia | *n* = 1 |
